# Risk of lung cancer among women in relation to lifetime history of tobacco smoking: a population-based case-control study in France (the WELCA study)

**DOI:** 10.1186/s12885-021-08433-z

**Published:** 2021-06-16

**Authors:** Jennifer Rusmaully, Nastassia Tvardik, Diane Martin, Régine Billmann, Sylvie Cénée, Martine Antoine, Hélène Blons, Pierre Laurent-Puig, Jean Trédaniel, Marie Wislez, Isabelle Stücker, Pascal Guénel, Loredana Radoï

**Affiliations:** 1 Université Paris-Saclay, UVSQ, Inserm U1018, CESP, Team Exposome and Heredity, Villejuif, France; 2 AP-HP, Tenon Hospital, Pathology, 4 rue de la Chine, Paris, France; 3 UPMC Univ Paris 06, GRC No.04, Theranoscan, Paris, France; 4 Assistance Publique Hôpitaux de Paris, Paris Cancer Institute CARPEM, AP-HP.Centre - Université de Paris, Department of Biology Physiology and Genetics, Hopital Européen Georges Pompidou, Paris, France; 5 Centre de Recherche des Cordeliers, INSERM, CNRS SNC 5096, Sorbonne Université, Université de Paris, Paris, France; 6 Groupe Hospitalier Paris Saint Joseph, Université de Paris, Unité INSERM UMR-S 1124, Toxicologie, pharmacologie et signalisation cellulaire, Paris, France; 7 AP-HP.Centre - Université de Paris, Hôpital Cochin, Unité d’Oncologie Thoracique, Service de Pneumologie, Paris, France; 8 Centre de Recherche des Cordeliers, Université de Paris, UMRS 1138 « Complement, Inflammation and Cancer », Paris, France; 9 AP-HP Nord - Université de Paris, Hôpital Louis Mourier, UFR d’odontologie, Paris, France

**Keywords:** Epidemiology, Lung cancer, Histology, Women, Tobacco, Smoking patterns, Cigarette dependence

## Abstract

**Background:**

This study aims to provide new insights on the role of smoking patterns and cigarette dependence in female lung cancer, and to examine differences by histological subtype.

**Methods:**

We conducted a population-based case-control study in the great Paris area among women including 716 incident cases diagnosed between 2014 and 2017 and 757 age-matched controls. Detailed data on smoking history was collected during in-person interviews to assess intensity and duration of tobacco smoking, time since cessation, smoking habits (depth of smoke inhalation, use of filter, type of tobacco, and type of cigarettes) and Fagerström test for cigarette dependence. The comprehensive smoking index (CSI), a score modelling the combined effects of intensity, duration and time since quitting smoking was determined for each subject. Multivariable logistic regression models were fitted to calculate odds ratios (ORs) and their confidence intervals (95%CI) of lung cancer associated with smoking variables.

**Results:**

Lung cancer risk increased linearly with intensity and duration of tobacco smoking while it decreased with time since cessation, to reach the risk in never-smokers after 20 years of abstinence. The combined effect of intensity and duration of tobacco smoking was more than multiplicative (p-interaction 0.012). The OR in the highest vs the lowest quartile of CSI was 12.64 (95%CI 8.50; 18.80) (p-trend < 0.001). The risk of small cell or squamous cell carcinomas increased with the CSI more sharply than the risk of adenocarcinomas. Deep smoke inhalation, dark vs blond tobacco, conventional vs light cigarettes, and unfiltered vs filtered cigarettes, as well as having mixed smoking habits, were found to be independent risk factors. Having high cigarette addiction behaviours also increased the risk after adjusting for CSI.

**Conclusion:**

This study provides additional insights on the effects of tobacco smoking patterns on lung cancer risk among women.

**Supplementary Information:**

The online version contains supplementary material available at 10.1186/s12885-021-08433-z.

## Background

With an estimated 2.1 million new cases and 1.8 million deaths in 2018, lung cancer is the leading cause of cancer incidence and mortality worldwide [1]. The lung cancer burden differs by sex. In men, lung cancer is the most common cause of cancer and cancer-related death, whereas in women it is the third most frequently diagnosed cancer and the second leading cause of cancer death worldwide [1]. Incidence rates in France in 2018 were 50.5 and 23.2 per 100,000 person-years in men and women, respectively [2].

Due to the increase in smoking among women in recent decades, lung cancer incidence rates in women has been steadily increasing and are converging with incidence rates among men [1]. In France, the increase of smoking prevalence among women from 20% in 1975 to over 30% in 2014 [3], resulted in a sharp increase in lung cancer incidence (+ 5% per year in 2010–2018), while the incidence rates among men were relatively stable over the same period (− 0.3% per year in 2010–2018) [2]. In the United States, where the smoking epidemic among women started earlier than in France, the patterns of historically higher incidence rates of lung cancer among men than among women have reversed in the younger generation born since the mid-1960s [4].

Historically, the most common histological subtype of lung cancer was adenocarcinoma in women and squamous cell carcinoma in men. The predominance of adenocarcinoma in women was concomitant with lower tobacco consumption and a higher proportion of non-smokers than in men with lung cancer [5–11]. Since the early 1990s, the proportion of adenocarcinoma has increased in women and in men with lung cancer and it now exceeds all other histological subtypes in both sexes [12]. This increase in the proportion of adenocarcinomas among lung cancer patients has been considered a consequence of a decline in smoking, which was found to be consistently associated with a lower risk of lung adenocarcinoma than squamous cell or small cell carcinoma [13–17]. It has also been suggested that the increasing proportion of adenocarcinoma among lung cancer patients might be due to changes in smoking behaviours and type of cigarette (i.e., lower tar and nicotine content of cigarettes, and increasing use of filter cigarettes) leading to deeper inhalation of tobacco smoke and more extensive exposure of peripheral lung tissue to small particles, prone to the development of adenocarcinomas [18, 19]. However, the use of a filter cigarette, the inhalation pattern or the type of tobacco smoked were not found to differ by histological subtype in a previous study among French women [17]. Thus, the role of smoking patterns on risk by histological subtype of lung cancer among women requires clarification.

It has also been suggested that the risk of lung cancer increases with intensity and duration of tobacco smoking more rapidly in women than in men [14, 16], possibly indicating that women have greater susceptibility than men to the carcinogenic effects of tobacco smoke [16, 20–22]. Specific features of the respiratory system in women, such as smaller lung size and differences in immunological and hormonal behaviours of the airways, could lead to a greater predisposition to lung cancer in female smokers [23]. However, case-control [24, 25] and prospective cohort studies [26, 27] as well a recent meta-analysis [28] have not found evidence that female smokers were at higher risk of lung cancer than male smokers with similar tobacco consumption.

In this population-based case-control study in France, we studied the role of duration, intensity, and time since quitting smoking on lung cancer risk among women, by considering each of these parameters separately or by examining their combined effect by using a comprehensive smoking index. We also compared the impact of smoking history on the major histological subtypes of lung cancer, and investigated the effects of smoking habits and cigarette addictive behaviours on risk.

## Methods

### Study population

The WELCA study (Women Epidemiology Lung Cancer) is a multicentre population-based case-control study conducted between 2014 and 2017 in the Ile-de-France region (great Paris area), subdivided in 8 ‘départements’ (administrative areas) [29].

Eligible cases were women aged 18 to 75 years newly diagnosed with primary cancer of the lung [ICD 10th revision [30] codes C34.0-C34.9] or trachea [ICD 10th revision code C33] in pneumology and oncology departments of public hospitals. All histological types were considered, except carcinoid tumours. From 849 eligible cases identified in the participating centres, 47 refused or were too ill to participate, 28 died before the interview, and 27 could not be contacted for an in-person interview. In addition, 31 cases responded a summary questionnaire and were excluded because details on tobacco smoking history and cigarette dependence were missing. In total, the present analysis included 716 cases (participation 84.3%).

Controls were women recruited from the general population in Ile-de-France and selected at random from the telephone book with the help of a polling institute. Controls were frequency-matched to the cases by age and ‘département’. In order to minimize selection bias that may arise from differential response rate across categories of socio-economic status (SES), quotas by SES were applied for the control group in order to reflect the distribution by SES of women of the same age in the general population. From 1107 eligible controls contacted by phone, 256 refused to participate, 67 could not be reached for an in-person interview, and 22 were too ill to participate. In addition, 4 controls who responded to the summary questionnaire and 1 control who refused to answer questions on tobacco smoking were excluded. In total, 757 controls were available for the present study (participation 68.3%).

Each subject gave an informed consent. The WELCA study was approved by the Institutional Review Board of the French National Institute of Health and Medical Research and by the French data Protection Authority (IRB-Inserm, no. 3888 and CNIL no. C13–52).

### Data collection

In-person interviews were conducted by trained research nurses using a standardised questionnaire administered by means of a Computer-Assisted Personal Interview (CAPI). We elicited information on socio-demographic characteristics, reproductive and hormonal history, anthropometric characteristics, personal and family medical history, lifetime residential and occupational history, lifestyle-related factors (tobacco smoking, alcohol drinking, anthropometric data and recreational physical activity).

For each smoking period defined by years of start and end of tobacco smoking, study participants were asked to report the mean number of cigarettes, cigarillos, or cigars smoked per day, type of tobacco smoked, i.e. blond tobacco (flue-cured tobacco produced by washing the tobacco leaves to reduce the amount of nicotine) or dark tobacco (air-cured tobacco with distinct content of nicotine and tars that used to be commonly smoked in France), type of cigarettes (light and/or classic), use of a filter (yes and/or no), and depth of smoke inhalation (no inhalation, shallow and/or deep). To determine the cigarette dependence according to the score of Fagerström et al. [31], participants were asked to answer 6 questions about the time at first cigarette after awakening (within 5 min, 6–30 min, 31–60 min, after 60 min), difficulty to refrain for smoking in places where it is forbidden (yes/no), essential cigarette in the day (the first one in the morning, another one, all, none), mean smoking intensity during dependence periods (≤10, 11–20, 21–30, ≥31 cigarettes/day), smoking intensity during the first hour after awakening compared to the rest of the day (higher/lower), and smoking while ill in bed most of the day (yes/no).

Data on tobacco smoking history were considered in the analysis up to a reference date defined as the date of diagnosis for the cases and the date of interview for the controls. The amount of tobacco smoked per day was converted into cigarette-equivalent using the following conversion factors: 1 cigarillo = 2 cigarettes and 1 cigar = 4 cigarettes based on the tobacco content in grams in one cigarette (1 g), one cigar (4 g) and one cigarillo (2 g) [32]. For simplicity, “cigarettes” will be used throughout the paper to refer to the amount of tobacco smoked. Smoking status was categorized as never smokers (women who smoked less than 100 cigarettes over the lifetime), former smokers (women who quit smoking for at least 2 years before reference date) and current smokers. Duration of smoking was computed as the sum of periods of tobacco smoking over the lifetime. Intensity of tobacco smoking was calculated as the mean number of cigarettes smoked per day, weighted on the duration of each period of tobacco smoking. Time since cessation was calculated as the number of years between the date of quitting smoking and the reference date. Pack-years was the number of packs of cigarettes or equivalent smoked per day (1 pack = 20 cigarettes) multiplied by the number of years of smoking.

We calculated the Comprehensive Smoking Index (CSI) that integrates intensity, duration, and time since smoking cessation into a single variable (see Supplementary material 1). This index was developed by Hoffmann et al. [33, 34] and adapted to lung cancer by Leffondré et al. [35]. The CSI was used in the present study to summarize the most relevant aspects of tobacco smoking history on lung cancer risk into a single score, with the advantage of avoiding multicollinearity between smoking variables in the statistical models. In particular, it was used as an adjustment covariate in the models that aimed to assess the effect of smoking behaviours and nicotine dependence on lung cancer risk.

### Statistical analysis

All smoking variables were considered as categorical using the following categories: intensity (never smokers, < 10, 10–19, 20–29, ≥30 cigarettes/day); duration (never smokers, < 20, 20–29, 30–39, ≥40 years); time since cessation (never smokers, current smokers, 2–9, 10–19, ≥20 years); CSI (quartiles of the distribution among cases and controls); inhalation of tobacco smoke (no inhalation, shallow, deep, mixed); type of tobacco smoked (blond, dark, mixed); use of a filter (yes, no, mixed) and type of cigarettes (light, classic, mixed). The score for cigarette dependence was calculated by summing the scores attributed to each of the 6 items of the Fagerström test for cigarette dependence. It ranges from 0 to 10, with 10 representing the highest level of dependence. This score was categorized as follows: 0–3 = low dependence; 4–6 = moderate dependence; 7–10 = high dependence. Associations with each of the six Fagerström cigarette addiction variables were also assessed separately to search for a dominant factor driving the association with the total score. All quantitative variables for tobacco smoking (intensity, duration, time since cessation and CSI) were also used as continuous variable in the models.

We calculated odds ratios (ORs) for lung cancer and their 95% confidence intervals (95% CI) associated with each smoking variable using unconditional logistic regression models, adjusted for the matching variables age (continuous) and ‘département’ (8 “départements” of the Ile-de-France region, see Table 1), as well as education (elementary school, middle school, high school, university) and body mass index (BMI) 2 years before the interview (< 18.5 kg/m2; 18.5–24.9 kg/m2; 25–29.9 kg/m2; ≥30 kg/m2). Additional adjustment for marital status, chronic respiratory diseases, and physical activity were also conducted but did not change the OR estimates and are not shown.

**Table 1:** Main characteristics of study population

	Cases ***N*** = 716 (%)	Controls ***N*** = 757 (%)	p
**AGE**			
< 50	95 (13.3)	86 (11.4)	
[50–60[	196 (27.4)	175 (23.1)	
[60–70[	309 (43.2)	353 (46.6)	
≥ 70	116 (16.2)	143 (18.9)	0.10
*mean (SD)*	*60.9 (9.4)*	*61.9 (9.5)*	*0.79*
**AREA OF RESIDENCE (‘Département’)**			
75 Paris	289 (40.4)	315 (41.6)	
77 Seine-et-Marne	27 (3.8)	22 (2.9)	
78 Yvelines	32 (4.5)	30 (4.0)	
91 Essonne	24 (3.4)	24 (3.2)	
92 Hauts-de-Seine	116 (16.2)	124 (16.4)	
93 Seine-Saint-Denis	84 (11.7)	73 (9.6)	
94 Val-de-Marne	130 (18.2)	156 (20.6)	
95 Val-d’Oise	14 (2.0)	13 (1.7)	0.78
**EDUCATION**			
University	336 (47.1)	372 (49.1)	
High school	117 (16.4)	141 (18.6)	
Middle school	199 (27.9)	211 (27.9)	
Elementary school or less	62 (8.7)	33 (4.4)	0.01
**BMI 2 years BEFORE THE INTERVIEW (kg/m**^**2**^**)**			
< 18.5	62 (8.7)	37 (4.9)	
[18.5–25.0[	402 (56.2)	350 (46.3)	
[25.0–30.0[	161 (22.5)	207 (27.4)	
≥ 30.0	90 (12.6)	162 (21.4)	< 0.001
*mean (SD)*	*24.2 (5.3)*	*25.8 (5.7)*	*0.03*
**HISTOLOGIC TYPES**	**Cases N (%)**		
Adenocarcinoma	514 (71.8)	–	
Squamous cell carcinoma	67 (9.4)	–	
Large cell carcinoma	28 (3.9)	–	
Small cell carcinoma	88 (12.3)	–	
Others	19 (2.6)	–	

Abbreviations: *BMI* body mass index; *SD* standard deviation

Descriptive analysis were drawn using the Student test for continuous variables and the X^2^ Pearson test for categorical variables

Trend tests between categorical variables for tobacco smoking and lung cancer risk were performed by introducing in the models the median values of each category as a quantitative variable. Interaction between intensity and duration of tobacco smoking was performed by adding and testing the significance of a multiplicative term in the logistic regression model using the maximum likelihood ratio test.

We also studied the association between all tobacco variables and the risk of the main histological types of lung cancer (adenocarcinoma, squamous cell carcinoma, and small cell carcinoma) using polytomous (multinomial) logistic regression models. The ORs for the different histological types were compared using the OR homogeneity test.

Statistical analyses were conducted using SAS (Statistical Analysis Software 9.4, SAS Institute Inc., Cary, North Carolina, USA).

## Results

### Selected characteristics of the study population

The main characteristics of the study population are shown in Table 1. As expected, the age distribution of the cases and the controls was similar with a mean age at 60.9 and 61.9 years, respectively. More than 40% of the women in both groups were residing in Paris. Cases were slightly less educated than the controls (*p* = 0.01) and had lower BMI two years before the interview (*p* = 0.03). Among cases, adenocarcinoma was the most frequent histological type (71.9%), followed by small cell carcinoma (12.3%) and squamous cell carcinoma (9.4%).

### Lung cancer risk related to smoking variables

Table 2 presents the ORs for lung cancer associated with tobacco smoking. When compared to never smokers, the OR for lung cancer was 2.15 for former smokers and 5.22 for current smokers. A linear trend was observed between the OR for lung cancer and the number of cigarettes per day, years of tobacco smoking, and pack-years. Lung cancer risk decreased with time since cessation of tobacco smoking and was close to unity (OR = 0.97; 95%CI 0.66; 1.42) in women who quit smoking for 20 or more years. When looking at the CSI accounting for the combined effects on lung cancer risk of intensity, duration and time since cessation, the OR increased more than 12-fold in the highest as compared to the lowest exposure quartile (p trend < 0.001).

**Table 2:** Associations between smoking variables and lung cancer

	Cases N = 716 (%)	Controls N = 757 (%)	OR (95%CI) ^a^	p trend
**SMOKING STATUS** ^b^				
Never smokers	142 (19.8)	341 (45.1)	Ref	
Former smokers	201 (28.1)	246 (32.5)	2.15 (1.62; 2.84)	
Current smokers	373 (52.1)	170 (22.5)	5.22 (3.96; 6.89)	
**INTENSITY, cigarettes/day**				
mean (SD)*	*18.5 (8.9)*	*12.5 (9.2)*		
Never smokers	142 (19.8)	341 (45.1)	Ref	
< 10	80 (11.2)	175 (23.2)	1.11 (0.79; 1.56)	
10–19	286 (39.9)	150 (19.8)	4.69 (3.52; 6.26)	
20–29	148 (20.7)	72 (9.5)	5.40 (3.78; 7.70)	
≥ 30	60 (8.4)	18 (2.4)	9.63 (5.37; 17.24)	< 0.001
*Per 10 cigarettes/day*			*2.27 (2.02; 2.56)*	
**DURATION OF TOBACCO SMOKING, years**				
mean (SD)*	*36.5 (11.6)*	*26.4 (14.8)*		
Never smokers	142 (19.8)	341 (45.1)	Ref	
< 20	62 (8.7)	150 (19.8)	1.07 (0.74; 1.55)	
20–29	74 (10.3)	76 (10.1)	2.19 (1.49; 3.23)	
30–39	184 (25.7)	94 (12.4)	4.80 (3.46; 6.65)	
≥ 40	254 (35.5)	95 (12.6)	7.43 (5.36; 10.30)	< 0.001
*Per 10 years*			*1.59 (1.49; 1.70)*	
**PACK-YEARS**				
mean (SD)*	*35.4 (21.0)*	*18.5 (17.5)*		
Never smokers	142 (19.8)	341 (45.1)	Ref	
< 10	57 (8)	173 (22.9)	0.81 (0.56; 1.17)	
10–19	83 (11.6)	86 (11.4)	2.40 (1.66; 3.48)	
20–29	106 (14.8)	58 (7.7)	4.37 (2.97; 6.44)	
≥ 30	328 (45.8)	98 (13.0)	9.16 (6.68; 12.54)	< 0.001
*Per 10 pack-years*			*1.65 (1.54; 1.76)*	
**TIME SINCE TOBACCO SMOKING CESSATION, years**				
mean (SD)*	*11.7 (10.7)*	*24.0 (14.6)*		
Never smokers	142 (19.8)	341 (45.1)	Ref	
Current smokers	373 (52.1)	170 (22.5)	5.30 (4.02; 7.00)	
2–9	84 (11.7)	45 (5.9)	4.78 (3.14; 7.27)	
10–19	65 (9.1)	58 (7.7)	2.78 (1.84; 4.20)	
≥ 20	52 (7.3)	143 (18.9)	0.97 (0.66; 1.42)	< 0.001
*Per 1 year (among ever smokers)*			*0.94 (0.93; 0.96)*	
**CSI** ^c^				
mean (SD)*	*1.6 (0.6)*	*1.0 (0.7)*		
Never smokers	142 (19.8)	341 (45.1)	Ref	
< 0.69	67 (9.4)	181 (23.9)	0.98 (0.69; 1.39)	
0.69–1.45	130 (18.2)	116 (15.3)	2.71 (1.95; 3.75)	
1.46–1.95	173 (24.2)	74 (9.8)	5.69 (4.02; 8.04)	
≥ 1.96	204 (28.5)	44 (5.8)	12.64 (8.50; 18.80)	< 0.001
*Per 1 CSI unit*			*3.13 (2.71; 3.61)*	

Abbreviations: *CI* confidence interval; *CSI* comprehensive smoking index; *OR* odds ratio; *SD* standard deviation

* Mean and SD were calculated among ever smokers using the Student test

^a^ ORs were adjusted for age (continuous), area of residence, education, and BMI 2 years before the interview (continuous)

^b^ Ever smoker correspond to a person who have smoked at least 100 cigarettes throughout life; former smoker correspond to a person who have stopped smoking for at least 2 years before the interview (or before the diagnosis for cases)

^c^ Quartiles among controls and cases

Table 3 shows the odds ratios associated with the variable combining duration and intensity among ever-smokers, using the group of “light” smokers (< 10 cigarettes/day) for “short” duration (< 20 years) as the reference category. In this table, the odds ratio in heavy smokers (≥20 cig/d) for a long duration (≥40 years) was as high as 19.02 (95%CI 9.66–37.46). The *p*-value for interaction between intensity and duration was 0.012, indicating that the combined effect of intensity and duration was greater than multiplicative. The odds ratios associated with the combined variable are also shown in supplementary Table S2 using each combination of duration and intensity in turn as a reference category. It can be seen that the odds ratios at any level of smoking intensity increased with smoking duration, regardless of the reference category. Similarly, the odds ratios for 20–39 years or ≥ 40 years of smoking duration increased with smoking intensity, regardless of the reference category. However, among short-duration smokers (< 20 years), the odds ratio for smoking intensity ≥20 cig/day did not increase noticeably when compared to smokers of 10–19 cig/day (OR _≥20 cig/d and < 20 years vs 10–19 cig/d and < 20 years_ = 0.84 (95% CI, 0.36; 1.96)), suggesting that smoking intensity beyond 10 cig/day had no or only weak additional impact on lung cancer risk among short-duration smokers.

**Table 3:** Combined effects of smoking intensity and smoking duration on lung cancer among ever smokers

EVER SMOKERS ^b^	INTENSITY (cigarettes/day)					
	< 10		10–19		≥20	
	Cases/Controls	OR (95%CI) ^a^	Cases/Controls	OR (95%CI) ^a^	Cases/Controls	OR (95%CI) ^a^
**DURATION (years)**						
**< 20**	23/81	1.00 (Ref)	26/41	2.08 (1.03; 4.19)	13/28	1.74 (0.76; 3.99)
**20–39**	36/65	1.62 (0.86; 3.07)	134/65	6.17 (3.50; 10.89)	88/40	7.50 (4.06; 13.87)
**≥ 40**	21/29	2.47 (1.16; 5.26)	126/44	10.26 (5.61; 18.75)	107/22	19.02 (9.66; 37.46)
p _interaction_ intensity*duration	0.012					

Abbreviations: *CI* confidence interval, *OR* odds ratio

^a^ Odds ratios adjusted for age (continuous), area of residence, education, and BMI 2 years before the interview (continuous)

^b^ Ever smoker correspond to a person who have smoked at least 100 cigarettes throughout life

### Histologic subtypes

Table 4 shows ORs associated with smoking variables on a continuous scale for the main histologic subtypes. Small cell carcinoma was the histologic subtype most strongly associated with smoking status, intensity, duration, pack-years, time since cessation, and CSI, followed by squamous cell carcinoma and adenocarcinoma. Paired comparisons between histologic subtypes were statistically significant regardless of the smoking variable.

**Table 4:** Association between smoking variables and histologic subtypes of lung cancer

	CONTROLS	ADENOCARCINOMA (1)		SQUAMOUS CELL CARCINOMA (2)		SMALL CELL CARCINOMA (3)		p-value for Homogeneity Test		
	N = 757	*N* = 514	OR (95%CI)	*N* = 67	OR (95%CI)	*N* = 88	OR (95%CI)	(1)/(2)	(1)/(3)	(2)/(3)
**SMOKING STATUS**										
Never smokers	341	129	Ref	7	Ref	3	Ref			
Former smokers	246	149	1.76 (1.31; 2.37)	24	5.23 (2.17; 12.59)	15	7.66 (2.17; 27.10)	0.15	0.89	0.43
Current smokers	170	236	3.50 (2.61; 4.70)	36	12.56 (5.33; 29.58)	70	59.43 (18.11; 194.97)	0.01	< 0.001	0.01
**INTENSITY**										
Per 10 cigarettes/day			2.04 (1.79; 2.31)		2.82 (2.23; 3.56)		3.45 (2.79; 4.28)	0.01	< 0.001	0.14
**DURATION**										
Per 10 years			1.44 (1.35; 1.54)		2.04 (1.71; 2.43)		2.83 (2.26; 3.55)	< 0.001	< 0.001	0.02
**PACK-YEARS**										
Per 10 pack-years			1.55 (1.45; 1.66)		1.86 (1.66; 2.09)		2.13 (1.90; 2.38)	< 0.001	< 0.001	0.04
**TIME SINCE CESSATION** *(among ever smokers)*										
Per 1 year			0.96 (0.94; 0.97)		0.92 (0.89; 0.95)		0.87 (0.82; 0.91)	0.03	< 0.001	0.05
**CSI**										
Per 1 CSI unit			2.54 (2.18; 2.96)		5.12 (3.61; 7.26)		9.98 (6.60; 15.09)	< 0.001	< 0.001	0.01

Abbreviations: *CI* confidence interval, *CSI* comprehensive smoking index, *OR* odds ratio

Odds ratio were adjusted for age (continuous), area of residence, education, and BMI 2 years before the interview (continuous)

### Lung cancer risk related to smoking patterns and cigarette dependence

The great majority of cases and controls smoked cigarettes (99%); only 3 cases (0.6%) and 2 controls (0.5%) smoked mixed cigarettes and cigarillos or cigarettes and cigars; 2 cases (0.3%) and 2 controls (0.5%) smoked cigarillos exclusively; no women smoked pipes. Therefore, we were not able to conduct analyses by type of product smoked.

Results on inhalation, type of tobacco, use of filter, and type of cigarettes are shown in Table 5. Before adjustment for CSI, the ORs for lung cancer were increased in inhalers, particularly in deep and mixed shallow and deep inhalers vs non-inhalers; in non-users and mixed users of filtered cigarettes vs users; in dark tobacco and mixed dark/blond tobacco smokers vs blond tobacco smokers; and in classic cigarette or mixed light/classic cigarette smokers vs light cigarette smokers. Odds ratios associated with inhalation, use of filter, type of tobacco, and type of cigarettes decreased after adjustment for CSI but remained elevated suggesting an independent effect of the smoking patterns on lung cancer risk.

**Table 5:** Associations between smoking patterns and lung cancer among ever smokers

	CASES ***N*** = 574 (%)	CONTROLS ***N*** = 416 (%)	OR (95%CI) ^a^	OR (95%CI) ^b^
**SMOKE INHALATION**				
No inhalation	46 (8.1)	73 (17.8)	Ref	Ref
Shallow	130 (22.8)	129 (31.4)	1.57 (0.98; 2.50)	1.02 (0.61; 1.73)
Deep	274 (48.0)	159 (38.7)	2.57 (1.65; 4.00)	1.22 (0.73; 2.03)
Mixed	121 (21.2)	50 (12.2)	3.70 (2.20; 6.21)	1.60 (0.90; 2.85)
**FILTER USE**				
Yes	435 (75.9)	348 (84.3)	Ref	Ref
No	33 (5.8)	26 (6.3)	1.02 (0.58; 1.80)	1.41 (0.74; 2.69)
Mixed	105 (18.3)	39 (9.4)	2.33 (1.54; 3.51)	1.57 (1.00; 2.44)
**TYPE OF TOBACCO**				
Blond	289 (50.5)	247 (60.1)	Ref	Ref
Dark	69 (12.1)	50 (12.2)	1.36 (0.88; 2.09)	1.47 (0.89; 2.40)
Mixed	214 (37.4)	114 (27.7)	1.74 (1.28; 2.37)	1.11 (0.79; 1.57)
**TYPE OF CIGARETTE**				
Light	38 (6.6)	50 (12.2)	Ref	Ref
Classic	373 (65.0)	278 (67.8)	1.89 (1.18; 3.02)	1.44 (0.87; 2.38)
Mixed	163 (28.4)	82 (20.0)	2.85 (1.70; 4.77)	1.70 (0.98; 2.96)

Abbreviations: *CI* confidence interval, *OR* odds ratio

^a^ Odds ratio adjusted for age (continuous), area of residence, education, and BMI 2 years before interview (continuous)

^b^ Odds ratio adjusted for age (continuous), area of residence, education, BMI 2 years before interview (continuous), and the comprehensive smoking index (continuous)

Table 6 shows that lung cancer risk was strongly associated with time to first cigarette after awakening. Women who smoked their first cigarette within 5 min after awakening were 5 times as likely to develop lung cancer as those who smoked their first cigarette after 60 min. Difficulty to refrain from smoking in places where it is forbidden, having essential cigarette in the day, and smoking while ill in bed showed a positive association with lung cancer that persisted after adjustment for the CSI. Odds ratios for lung cancer increased dramatically with the overall Fagerström dependence score and remained elevated after adjustment for the CSI, suggesting an independent effect of cigarette addiction on lung cancer risk.

**Table 6:** Associations between the Fagerström score for nicotine dependence (by item and overall) and lung cancer among ever smokers

	Cases N (%)	Controls N (%)	OR (95% CI) ^**a**^	OR (95% CI) ^**b**^
**Time at first cigarette after awakening**				
After 60 min	111 (19.5)	205 (50.0)	Ref	Ref
31 to 60 min	70 (12.3)	43 (10.5)	2.94 (1.86; 4.64)	1.61 (0.98; 2.64)
6 to 30 min	219 (38.4)	99 (24.2)	4.00 (2.83; 5.64)	1.72 (1.16; 2.57)
Within 5 min	170 (29.8)	63 (15.4)	5.21 (3.54; 7.66)	1.96 (1.25; 3.06)
**Difficulty to refrain for smoking in places where it is forbidden**				
No	389 (68.7)	346 (85.2)	Ref	Ref
Yes	177 (31.3)	60 (14.8)	2.68 (1.91; 3.75)	1.73 (1.21; 2.48)
**Essential cigarette in the day**				
None	56 (10.0)	118 (29.4)	Ref	Ref
Other than the first one	124 (22.1)	122 (30.4)	2.08 (1.37; 3.16)	1.23 (0.77; 1.94)
The first one in the morning	307 (54.8)	128 (31.9)	4.85 (3.28; 7.17)	1.79 (1.13; 2.83)
All	73 (13.0)	33 (8.2)	4.67 (2.74; 7.97)	1.93 (1.07; 3.48)
**Mean smoking intensity during dependence periods (cigarettes/day)**				
≤ 10	53 (9.3)	138 (33.6)	Ref	Ref
11–20	192 (33.5)	144 (35)	3.36 (2.26; 5.00)	1.42 (0.90; 2.24)
21–30	135 (23.6)	65 (15.8)	5.49 (3.51; 8.59)	1.93 (1.15; 3.24)
≥ 31	193 (33.7)	64 (15.6)	8.39 (5.4; 13.06)	2.30 (1.34; 3.93)
**Smoking intensity during the first hour after awakening compared to the rest of the day**				
Not different	358 (65.2)	343 (84.1)	Ref	Ref
More intense	191 (34.8)	65 (15.9)	2.60 (1.88; 3.61)	1.11 (0.76; 1.61)
**Smoking while ill in bed most of the day**				
No	256 (46.4)	293 (72.4)	Ref	Ref
Yes	296 (53.6)	112 (27.7)	2.96 (2.23; 3.94)	1.44 (1.04; 1.99)
**Overall total Fagerström score**				
0–3 (low dependence)	138 (27.0)	224 (59.6)	Ref	Ref
4–6 (moderate dependence)	173 (33.9)	90 (23.9)	3.15 (2.24; 4.44)	1.56 (1.06; 2.31)
7–10 (high dependence)	200 (39.1)	62 (16.5)	5.08 (3.52; 7.34)	1.93 (1.25; 2.99)

Abbreviations: *CI* confidence interval, *OR* odds ratio

^a^ Odds ratio adjusted for age (continuous), area of residence, education, and BMI 2 years before interview (continuous)

^b^ Odds ratio adjusted for age (continuous), area of residence, education, BMI 2 years before interview (continuous), and the comprehensive smoking index (continuous)

Results on inhalation, type of tobacco, use of filter, type of cigarettes and overall cigarette dependence score by histologic subtype are shown in Supplementary Table S1. With the exception of an increased OR for dark vs blond tobacco in squamous cell carcinoma (OR = 3.72; 95%CI 1.58; 8.77), no clear difference between lung cancer subtypes was observed in relation to smoking behaviours and cigarette dependence score, but these findings are based on small numbers.

## Discussion

This study provides new insights on the relationship between tobacco smoking history and lung cancer in women. As expected, a clear dose-response relationship was observed between lung cancer risk and smoking intensity, smoking duration and number of pack-years. The risk declined gradually with time since quitting smoking and reached the risk of never smokers after 20 years. The exposure-risk relationship was particularly pronounced with the CSI score, which models the intricate effects of intensity, duration, and time since quitting. In addition, an interaction on the multiplicative scale between intensity and duration of tobacco smoking was observed. Adenocarcinoma was the histologic type least affected by tobacco smoking, and small cell lung cancer the one most affected. Deep smoke inhalation, use of dark rather than blond tobacco, conventional rather than light cigarettes, unfiltered rather than filtered cigarettes, or having mixed smoking habits as well as high cigarette addiction behaviours, may further increase the risk of lung cancer in women.

### Duration, intensity and time since cessation of smoking

Most previous studies have exclusively relied on pack-years of tobacco smoking as the main exposure metric to describe the relation between tobacco smoking over lifetime and lung cancer risk. However, this exposure metric is insufficient to characterise lifetime tobacco smoking history because it does not account for possible interaction between intensity and duration, and does not consider time since quitting smoking. We found that intensity and duration of smoking were distinct predictors of lung cancer risk in women, i.e., that lung cancer risk increased with the number of cigarettes smoked per day in women who smoked less than 20 years (“short-term” smokers), and similarly that it increased with the number of years of smoking in women who smoked less than 10 cigarettes per day (“light smokers”). In addition, we found that lung cancer risk associated with exposure to both high intensity and long duration of tobacco smoking was stronger than the multiplication of the effects of each parameter separately, i.e. that there was an interaction on the multiplicative scale. It was also suggested that smoking intensity beyond 10 cig/day had no further impact on lung cancer risk among short-duration smokers (Table S2).

In addition, to account for the intricate effects of duration, intensity, and time since quitting tobacco smoking, we calculated the CSI that provides a relevant measure of tobacco smoking over lifetime. By integrating the most important characteristics of smoking history into a single score, the CSI avoids problems of multicollinearity between duration, intensity and time since quitting smoking [35]. We found a clear dose-response trend between the CSI and the lung cancer risk indicating that this score is strongly predictive of lung cancer risk.

### Histological subtypes

Similar to previous studies among women [14–17], we found that tobacco smoking was more weakly associated with adenocarcinomas than with squamous cell or small cell carcinomas. Indeed, in our study the odds ratio per one-point increment of the CSI was 2.0 and 3.9 higher for squamous cell and small cell carcinoma than for adenocarcinomas, respectively. This gradient was also reflected by the high proportion of never smokers among adenocarcinoma cases (25.1%), in comparison to squamous cell (10.5%) or small cell carcinomas (3.4%).

Adenocarcinoma has been the predominant lung cancer subtype among female patients (e.g. 72% in our study). In addition, the predominance of adenocarcinoma in female lung cancer patients has become more pronounced over time, since adenocarcinoma incidence rates in women increased during the last decades (e.g. + 7.7% per year on average between 1990 and 2018 in France [2] and in the USA [36]), while incidence rates of other histological types decreased [2]. However, the relatively moderate tobacco consumption and the recent decrease of smoking prevalence among women in France [32] may not be sufficient to explain the large and increasing predominance of adenocarcinomas in female lung cancer patients. Changes in cigarette design and composition may also explain part of the elevated prevalence of adenocarcinomas among women [16]. The introduction of filter cigarettes could be responsible for deeper inhalation of small carcinogenic particles into the distal airways, resulting in changes in the histological type and anatomic location of lung cancer, i.e., promoting adenocarcinomas with a more peripheral distribution. Changes in the composition of cigarettes, with lowered tar and nicotine content but increased nitrosamine concentrations, could also contribute to this evolution [16, 37, 38].

In addition to smoking behaviors, other lung cancer risk factors could play a role and promote the development of adenocarcinomas. An association between exposure to environmental tobacco smoke and lung adenocarcinoma with dose-response relationship has been demonstrated among female non-smokers [39]. Air pollution and particulate matter (PM10, PM2.5) have been recognized as carcinogenic to humans by IARC [40] and the association with lung cancer was found to be stronger for adenocarcinoma [41]. Hormonal and reproductive factors in women have also been associated with lung cancer risk with stronger relationships for adenocarcinoma [42]. To investigate further risk factors for lung cancer other than smoking, epidemiological studies should be conducted for specific histological subtypes. In these studies, fine-grained information on the role of smoking habits by histology, as provided here, will be needed to be used as adjustment or modification factors in the analyses.

### Smoking habits, type of tobacco and dependence

We found that deep inhalation increased risk of lung cancer although the odds ratios were no longer significant after adjusting for the CSI. This result should be seen with caution, since self-reporting of inhalation patterns may not adequately reflect cotinine levels [43].

Similarly, we found that using non-filtered (or both filtered and non-filtered) vs filtered cigarettes, smoking dark tobacco (or both dark and blond tobacco) vs blond tobacco only, and smoking classic (or both light and classic cigarettes) vs light cigarettes only, were associated with increased odds ratios for lung cancer, although the associations were weaker and sometimes non-significant after adjusting for the CSI.

With regard to type of tobacco smoked, a greater impact of dark tobacco is plausible because the levels of carcinogenic DNA-adducts were found to be 1.5 times greater and excretion of urinary mutagens 1.8 times higher in smokers of dark tobacco than in smokers of blond tobacco [44]. Similar to the study of Papadopoulos et al. [17], we found no difference in relation to tobacco smoking patterns between histological types of lung cancer, except for the use of dark tobacco which was significantly associated with the risk of squamous cell carcinoma. However, the findings by histological subtypes are based on small numbers, preventing firm conclusions.

Cigarette dependence was associated with an increased risk of lung cancer for each addiction item of the Fagerström test and for the total score. The associations were still apparent after adjusting for CSI, thus controlling for intensity, duration and time since quitting smoking, indicating that cigarette addiction may be an independent risk factor for lung cancer. Similar findings were observed in two case-control studies [45, 46]. Gu et al. mainly focused on time to first cigarette [46] and reported an increased risk of lung cancer with shorter time to first cigarette. Thomas et al. [45] found that lung cancer risk increased with overall Fagerström dependence score as well as with each of its components, except for addiction variable related to smoking while ill in bed. In both studies, the associations between cigarette dependence and lung cancer were strongest in patients with squamous cell carcinomas.

Nicotine, the active ingredient in tobacco, is the primary contributing factor for dependence [44]. Although nicotine is not considered carcinogenic, in vitro studies have shown that it may contribute to cancer development by several mechanisms: disruption of cell signalling pathways, promotion of cellular proliferation and endothelial cell migration, increase of angiogenic growth factors and decrease of tumour suppression [44]. However, the role of cigarette dependence as an independent risk factor for lung cancer still requires confirmation.

### Study strengths and limitations

The main strengths of this study include the large female sample size and the availability of detailed information about different smoking exposure metrics, smoking patterns, and cigarette dependence to characterize the tobacco smoking throughout life for each subject, overall and by histological subtype. Potential recall and observer bias were minimized using a standardized questionnaire administered by trained research nurses who conducted interview for both cases and controls under identical conditions. The detailed and varied parameters on lifelong tobacco smoking has made it possible to carry out fine analyses on this lifestyle habit, especially to construct an interesting and useful tool, the CSI.

Cases were recruited in Paris pneumology and oncology departments of public hospitals. Implementing the study in a densely populated area enabled to include the major clinical wards that treat lung cancer patients, and to optimize the number of eligible female lung cancer patients. The treating physicians solicited their patients to participate, and face to face interviews with trained interviewers were conducted shortly after diagnosis in order to reduce the potential survival bias. For recruiting the controls, we applied quotas by socioeconomic status (SES) to minimize selection bias that may arise from differential participation rates by SES category. Although selection bias of cases and controls cannot be excluded, high participation rates were obtained for both cases and controls.

This study was retrospective in design making it prone to recall bias. Moreover, as tobacco use is a well-known risk factor for lung cancer, it is possible that female patients voluntarily minimized their consumption, resulting in a differential recall bias and underestimates of risk. In a national survey on tobacco smoking in France conducted in 2017, it was shown that 28.7% of French women were current smokers with an average daily consumption of 12.2 cigarettes, 27.7% were former smokers and 43.6% were never smokers [32]. These percentages were similar to those reported by controls in our study and were reassuring on the representativeness of the control group (22.5% current smokers with an average daily consumption of 12.5 cigarettes, 32.5% former smokers and 45% never smokers). Finally, we did not consider possible confounders in the analysis such as occupational exposures [47–49], diet [50, 51], or exposure to domestic fuel or cooking oil fumes [52], but a strong confounding effect is very unlikely.

## Conclusion

This study provides new highlights on lung cancer risk and smoking habits in women. Intensity, duration and time since quitting are the main predictors, but the type of tobacco smoked, the use of filtered cigarettes and nicotine addiction appear to be independently associated with risk. The smoking patterns affect lung cancer risk to very different extent depending on the histology. Further studies on lifetime smoking patterns are needed to better understand the time trends of lung cancer incidence among women and the variations by histological subtype.

## Supplementary Information

**Additional file 1 Supplementary material 1**: Calculation of the Comprehensive Smoking Index.

**Additional file 2 Supplementary Table S1**: Associations between smoking patterns, cigarette dependence and histologic types of lung cancer among ever smokers. **Supplementary Table S2**: Odds ratios and 95% confidence intervals for lung cancer among ever smokers associated with a variable combining intensity and duration of smoking, calculated using each category in turn as the reference (the WELCA study).

## Data Availability

The data that support the findings of this study are available from the corresponding author upon reasonable request.

## Abbreviations

BMI: Body mass index
CAPI: Computer assisted personal interview
CI: Confidence interval
CSI: Comprehensive Smoking Index
ICD 10th: International Classification of Diseases-10th revision
OR: Odds ratio
SES: Socio-economic status
SD: Standard deviation
