# Supplementary material for: Risk of lung cancer among women in relation to lifetime history of tobacco smoking: a population-based case-control study in France (the WELCA study)
Source: BMC Cancer. 2021 Jun 16;21:711. doi: 10.1186/s12885-021-08433-z (PMC8207748; doi:10.1186/s12885-021-08433-z)
Supplement: Supplementary file 1 — Additional file 1 Supplementary material 1: Calculation of the Comprehensive Smoking Index. [file 12885_2021_8433_MOESM1_ESM.docx]

**Supplementary material: Calculation of the Comprehensive Smoking Index - CSI - (according to Leffondré et al.)**

The formula to calculate the Comprehensive Smoking Index (CSI) from Leffondre et al. (1) is:

CSI = (1 − 0.5 ^dur*/τ^)(0.5^tsc*/τ^) ln(int + 1)]

where

tsc = time since smoking cessation

dur = duration of smoking

int = intensity of tobacco smoking

tsc* = max(tsc−δ, 0)

dur* = max(dur+tsc−δ, 0)−tsc*

The parameter δ describes the lag-time between ‘causal action’ and disease detection. This parameter accounts for the increase in lung cancer risk that has been observed in the first years after cessation.

The parameter τ can be interpreted as the duration of the health impact of smoking. It gives information on the form of the dose–response curve between duration (or time since cessation) and the risk of lung cancer.

To estimate the two parameters, we performed a series of logistic regression models, including the CSI as a continuous covariate. Each model contains a CSI calculated using a (τ, δ) pair selected from a dense two-dimensional grid. The τ parameter had a range of 13.1-52.2 years in 1-year increments, and the δ parameter had a range of 0.3-1.7 years in 0.1-year increments as found by Leffondre et al. in a female sample. The selected pair (τ=26 years, δ=0.7 years) corresponded to the model with the best goodness of fit, i.e. the model yielding the minimum Akaike’s Information Criterion (AIC).

**Reference**

1. Leffondre K, Abrahamowicz M, Xiao Y, Siemiatycki J. Modelling smoking history using a comprehensive smoking index: application to lung cancer. Stat Med. 2006;25(24):4132-4146.
