# Supplementary material for: Risk of lung cancer among women in relation to lifetime history of tobacco smoking: a population-based case-control study in France (the WELCA study)
Source: BMC Cancer. 2021 Jun 16;21:711. doi: 10.1186/s12885-021-08433-z (PMC8207748; doi:10.1186/s12885-021-08433-z)
Supplement: Supplementary file 2 — Additional file 2 Supplementary Table S1: Associations between smoking patterns, cigarette dependence and histologic types of lung cancer among ever smokers. Supplementary Table S2: Odds ratios and 95% confidence intervals for lung cancer among ever smokers associated with a variable combining intensity and duration of smoking, calculated using each category in turn as the reference (the WELCA study). [file 12885_2021_8433_MOESM2_ESM.docx]

Table S1. Odds ratios* of lung cancer by histologic subtype and smoking patterns or cigarette dependence among ever smokers (the WELCA study)

|  | **CONTROLS** | **ADENOCARCINOMA (1)** | | **SQUAMOUS CELL CARCINOMA (2)** | | **SMALL CELL CARCINOMA (3)** | | **p-value of OR Homogeneity Test** | | |
| --- | --- | --- | --- | --- | --- | --- | --- | --- | --- | --- |
|  | N=416 (%) | N=385 | **OR (95%CI)** | N=60 | **OR (95%CI)** | N=85 | **OR (95%CI)** | (1)/(2) | (1)/(3) | (2)/(3) |
| **SMOKE INHALATION** | | |  |  |  |  |  |  |  |  |
| No inhalation | 73 | 28 | Ref | 4 | Ref | 12 | Ref |  |  |  |
| Shallow | 129 | 88 | 1.12 (0.63; 2.00) | 9 | 0.97 (0.27; 3.54) | 22 | 0.56 (0.23; 1.40) | 0.32 | 0.73 | 0.27 |
| Deep | 159 | 183 | 1.35 (0.77; 2.36) | 33 | 2.23 (0.69; 7.22) | 37 | 0.48 (0.20; 1.13) | 0.17 | 0.18 | 0.03 |
| Mixed | 50 | 85 | 1.86 (0.99; 3.50) | 14 | 2.58 (0.73; 9.10) | 12 | 0.48 (0.18; 1.32) | 0.59 | 0.03 | 0.04 |
|  |  |  |  |  |  |  |  |  |  |  |
| **FILTER USE** |  |  |  |  |  |  |  |  |  |  |
| Yes | 348 | 302 | Ref | 41 | Ref | 58 | Ref |  |  |  |
| No | 26 | 16 | 0.96 (0.45; 2.02) | 6 | 2.75 (0.95; 7.94) | 7 | 2.57 (0.92; 7.21) | 0.06 | 0.08 | 0.87 |
| Mixed | 39 | 66 | 1.53 (0.96; 2.45) | 13 | 1.66 (0.76; 3.62) | 20 | 1.78 (0.90; 3.54) | 0.29 | 0.37 | 0.83 |
|  | |  |  |  |  |  |  |  |  |  |
| **TYPE OF TOBACCO** | |  |  |  |  |  |  |  |  |  |
| Blond | 247 | 212 | Ref | 17 | Ref | 35 | Ref |  |  |  |
| Dark | 50 | 35 | 1.09 (0.63; 1.89) | 14 | 3.72 (1.58; 8.77) | 12 | 1.77 (0.77; 4.07) | 0.01 | 0.24 | 0.25 |
| Mixed | 114 | 136 | 1.08 (0.75; 1.55) | 29 | 1.84 (0.91; 3.70) | 38 | 1.15 (0.64; 2.08) | 0.79 | 0.54 | 0.79 |
|  | |  |  |  |  |  |  |  |  |  |
| **TYPE OF CIGARETTE** | |  |  |  |  |  |  |  |  |  |
| Light | 50 | 27 | Ref | 2 | Ref | 7 | Ref |  |  |  |
| Classic | 278 | 248 | 1.56 (0.91; 2.70) | 44 | 2.02 (0.45; 9.07) | 51 | 0.60 (0.24; 1.55) | 0.36 | 0.12 | 0.07 |
| Mixed | 82 | 110 | 1.94 (1.07; 3.54) | 14 | 1.47 (0.30; 7.19) | 27 | 0.75 (0.27; 2.05) | 0.41 | 0.15 | 0.89 |
|  |  |  |  |  |  |  |  |  |  |  |
| **FAGERSTRÖM test for cigarette dependence** | | | |  |  |  |  |  |  |  |
| 0-2 | 196 | 74 | Ref | 5 | Ref | 7 | Ref |  |  |  |
| 3-6 | 118 | 151 | 1.84 (1.21; 2.82) | 24 | 3.67 (1.23; 10.97) | 30 | 2.35 (0.91; 6.03) | 0.31 | 0.93 | 0.45 |
| 7-10 | 62 | 121 | 2.12 (1.27; 3.52) | 21 | 4.07 (1.22; 13.53) | 39 | 3.24 (1.19; 8.83) | 0.45 | 0.37 | 0.99 |

Abbreviations: CI: confidence interval, OR: odds ratio.
^*^ All odds ratios are adjusted for age (continuous), area of residence, education, BMI 2 years before interview (continuous), and the comprehensive smoking index (continuous).

Table S2: Odds ratios* and 95% confidence intervals for lung cancer among ever smokers associated with a variable combining intensity and duration of smoking, calculated using each category in turn as the reference (the WELCA study)

|  | **INTENSITY (cig/day)** | | |
| --- | --- | --- | --- |
| **DURATION (years)** | **<10 cig/day** | **10-19 cig/day** | **≥20 cig/day** |
| **<20 years** | 1.00 (ref) | 2.08 (1.03 ; 4.19) | 1.74 (0.76 ; 3.99) |
| **20-39 years** | 1.62 (0.86; 3.07) | 6.17 (3.50; 10.89) | 7.50 (4.06; 13.87) |
| **≥40 years** | 2.47 (1.16; 5.26) | 10.26 (5.61; 18.75) | 19.02 (9.66; 37.46) |
| **<20 years** | 0.48 (0.24; 0.97) | 1.00 (ref) | 0.84 (0.36; 1.96) |
| **20-39 years** | 0.78 (0.40; 1.52) | 2.97 (1.63; 5.44) | 3.61 (1.89; 6.91) |
| **≥40 years** | 1.19 (0.54; 2.64) | 4.94 (2.58; 9.47) | 9.16 (4.47; 18.79) |
| **<20 years** | 0.57 (0.25; 1.31) | 1.19 (0.51; 2.79) | 1.00 (ref) |
| **20-39 years** | 0.93 (0.42; 2.07) | 3.54 (1.69; 7.45) | 4.30 (1.98; 9.37) |
| **≥40 years** | 1.42 (0.58; 3.47) | 5.89 (2.73; 12.71) | 10.92 (4.77; 24.98) |
| **<20 years** | 0.62 (0.33; 1.17) | 1.28 (0.66; 2.50) | 1.08 (0.48; 2.39) |
| **20-39 years** | 1.00 (ref) | 3.81 (2.26 ; 6.42) | 4.63 (2.60 ; 8.22) |
| **≥40 years** | 1.52 (0.74; 3.14) | 6.33 (3.59; 11.15) | 11.74 (6.15; 22.38) |
| **<20 years** | 0.16 (0.09; 0.29) | 0.34 (0.18; 0.62) | 0.28 (0.13; 0.59) |
| **20-39 years** | 0.26 (0.16; 0.44) | 1.00 (ref) | 1.22 (0.74; 1.99) |
| **≥40 years** | 0.40 (0.21; 0.78) | 1.66 (1.03; 2.69) | 3.08 (1.74; 5.46) |
| **<20 years** | 0.13 (0.07; 0.25) | 0.28 (0.15; 0.53) | 0.23 (0.11; 0.51) |
| **20-39 years** | 0.22 (0.12; 0.38) | 0.82 (0.50; 1.35) | 1.00 (ref) |
| **≥40 years** | 0.33 (0.16; 0.67) | 1.37 (0.80; 2.34) | 2.54 (1.37; 4.69) |
| **<20 years** | 0.41 (0.19; 0.87) | 0.84 (0.38; 1.87) | 0.71 (0.29; 1.73) |
| **20-39 years** | 0.66 (0.32; 1.36) | 2.50 (1.29; 4.87) | 3.04 (1.50; 6.17) |
| **≥40 years** | 1.00 (ref) | 4.16 (2.12 ; 8.17) | 7.71 (3.66 ; 16.24) |
| **<20 years** | 0.10 (0.05; 0.18) | 0.20 (0.11; 0.39) | 0.17 (0.08; 0.37) |
| **20-39 years** | 0.16 (0.09; 0.28) | 0.60 (0.37; 0.98) | 0.73 (0.43; 1.25) |
| **≥40 years** | 0.24 (0.12; 0.47) | 1.00 (ref) | 1.85 (1.03; 3.33) |
| **<20 years** | 0.05 (0.03; 0.10) | 0.11 (0.05; 0.22) | 0.09 (0.04; 0.21) |
| **20-39 years** | 0.09 (0.05; 0.16) | 0.32 (0.18; 0.58) | 0.39 (0.21; 0.73) |
| **≥40 years** | 0.13 (0.06; 0.27) | 0.54 (0.30; 0.97) | 1.00 (ref) |

* Odds ratios adjusted for age, département, education, and BMI 2 years before the interview.

**Reading examples**

- The odds ratios associated with smoking intensity increase with duration. For example:

ORs _≥20 vs <10 cig/d_ = 1.74, 4.63, 7.71 for duration <20 yrs, 20-39 yrs, and ≥40 yrs, respectively

- The odds ratios associated with smoking duration increase with intensity. For example:

ORs _≥40 yrs vs <20 yrs_ = 2.47, 4.94, 10.92 for intensity <10 cig/d, 10-19 cig/d, and ≥20 cig/d, respectively

- Among short duration smokers (<20 years), the OR associated with <10 cig/d is divided by 2 when compared to smokers of 10-19 cig/d (reference): OR _<10 cig/d vs 10-19 cig/d_ = 0.48 (95% CI, 0.24; 0.97)

- Among short duration smokers (<20 years), the OR associated with ≥20 cig/d does not differ from 1 when compared to smokers of 10-19 cig/d (reference): OR _≥20 cig/d vs 10-19 cig/d_ = 0.84 (95% CI, 0.36; 1.96)
